# Experiences With Intravenous Thrombolysis in Acute Ischemic Stroke by Elderly Patients–A “Real World Scenario”

**DOI:** 10.3389/fneur.2021.721337

**Published:** 2021-09-13

**Authors:** Máté Héja, István Fekete, László Horváth, Sándor Márton, Klára Edit Fekete

**Affiliations:** ^1^Department of Neurology, Faculty of Medicine, University of Debrecen, Debrecen, Hungary; ^2^Department of Neurology, Faculty of Medicine, University of Debrecen, Debrecen, Hungary; ^3^Department of Pharmaceutical Surveillance and Economics, Faculty of Pharmacy, University of Debrecen, Debrecen, Hungary; ^4^Institute of Political Science and Sociology, Faculty of Arts, University of Debrecen, Debrecen, Hungary; ^5^Department of Neurology, Faculty of Medicine, University of Debrecen, Debrecen, Hungary

**Keywords:** ischemic stroke, thrombolysis, elderly, outcome, symptomatic intracerebral hemorrhage

## Abstract

**Objectives:** This retrospective single-center study aimed to investigate the risk factors, outcomes and complication rates in patients older vs. younger than 80 years treated with intravenous alteplase.

**Methods:** Data of 1,253 thrombolysed patients were analyzed between January 1, 2004 and August 31, 2016. Vascular risk factors, stroke severity based on the NIHSS score, functional outcome using modified Rankin Scale (mRS), mortality and symptomatic intracerebral hemorrhage (SICH) were compared between two subgroups (<80 and ≥80 years).

**Results:** 1,125 patients were included, 199 (17.6%) among them were aged over 80 years, majority (63.3%) were female (*p* < 0.00001). Mean age was 68.2 ± 12.4 years, i.e., 64.7 ± 10.8 years and 84.3 ± 3.4 years in the younger and the older groups, respectively (*p* < 0.001). Atrial fibrillation and pre-stroke anticoagulation among patients over 80 years was more likely (*p* < 0.0005 and *p* = 0.02, respectively). NIHSS scores on admission and at 24 h were higher in elderly patients (*p* < 0.0001). ASPECT score at 24 h was less favorable in elderly patients (*p* = 0.007) and was associated with worse outcome. At 3 months, 59.8% of the patients from the older group had an unfavorable outcome (*p* < 0.0001), however 34.7% had independent outcome. The one-year- survival was significantly worse in the older group (*p* < 0.0001). The incidence of SICH was lower among older patients. In a logistic regression model, atrial fibrillation, heart failure, diabetes mellitus and smoking were proven as a significant independent risk factors for worse outcome.

**Conclusion:** Although, the outcomes were less favorable in patients over 80 years of age, our results support the feasibility of using intravenous thrombolysis among patients over 80 years of age.

## Introduction

Stroke is the second most common cause of death and a major cause of disability worldwide (1). According to the WHO statistics, 15 million people suffer a stroke in the world annually. Of these, 5 million die, and another 5 million are left permanently disabled, placing a burden on the family and community (2). Age is the most remarkable non-modifiable risk factor for stroke and a major predictor of clinical outcome (3). In fact, the incidence of stroke is rapidly increasing with age in both genders, doubling each decade after age 55 (4). Intravenous alteplase (recombinant tissue plasminogen activator [IV-rtPA]) is the only approved and validated treatment for pharmacological revascularisation in acute ischaemic stroke. However, despite the high prevalence of stroke in the elderly, data on the safety and efficacy of thrombolysis in the >80 years population were limited for long. In the first major IV-rtPA study, the NINDS trial, treatment of ischaemic stroke with alteplase was not specifically investigated in patients aged over 80, and in further studies, like ECASS-II, this population was excluded. This age restriction came from the potential higher risk of cerebral bleeding and caused uncertainty about the risk-benefit profile in these patients (5). Earlier studies seem to show that high age is an independent predictor of symptomatic intracerebral hemorrhage (SICH) in patients treated with IV-rtPA and the incidence of SICH increases with age (3, 6). However, more and more data support that patients from this age group still seem to benefit from this treatment. Several observational studies have demonstrated the conclusions above, though the outcome at 3 months has been worse for the older patients than for their younger counterparts, the elderly do not seem to have an increased risk for SICH after IV-rtPA (7–9). One of the largest controlled comparisons of SITS International Stroke Thrombolysis Registry and Virtual International Stroke Trials suggests that increasing age is associated with poorer outcome, but the association between thrombolysis treatment and improved outcome is maintained in very elderly people (10). The conclusion of all these studies is that age alone should not be the reason to exclude patients from treatment with IV-rtPA.

Altogether more than one-third of acute strokes occur among people aged ≥80 years, and due to increasing life expectancy the incidence of stroke will continue to rise in this age group. This problem particularly affects the Central-Eastern European countries where the stroke is more frequent, the mortality rate is higher, and the risk factors such as obesity, hypertension and alcohol abuse are more prevalent than is western Europe (11). However, intravenous alteplase is a safe and effective treatment for acute ischemic stroke within 4.5 h, until 2020, it was only approved for patients aged 18–80 years, and can be used in patients over 80 years on an individual benefit-risk basis (12). In order to select the most eligible elderly patients for thrombolysis, we need to identify the risk factors associated with worse outcome.

In the current work, we present a single-center report on patients over 80 years receiving rt-PA after acute ischaemic stroke. The aim of this study was to compare the risk factors, functional outcomes and complication rates in patients older vs. lower than 80 years old. A real-life scenario was conducted.

## Methods

### Subjects, Patients

**Figure 1:**
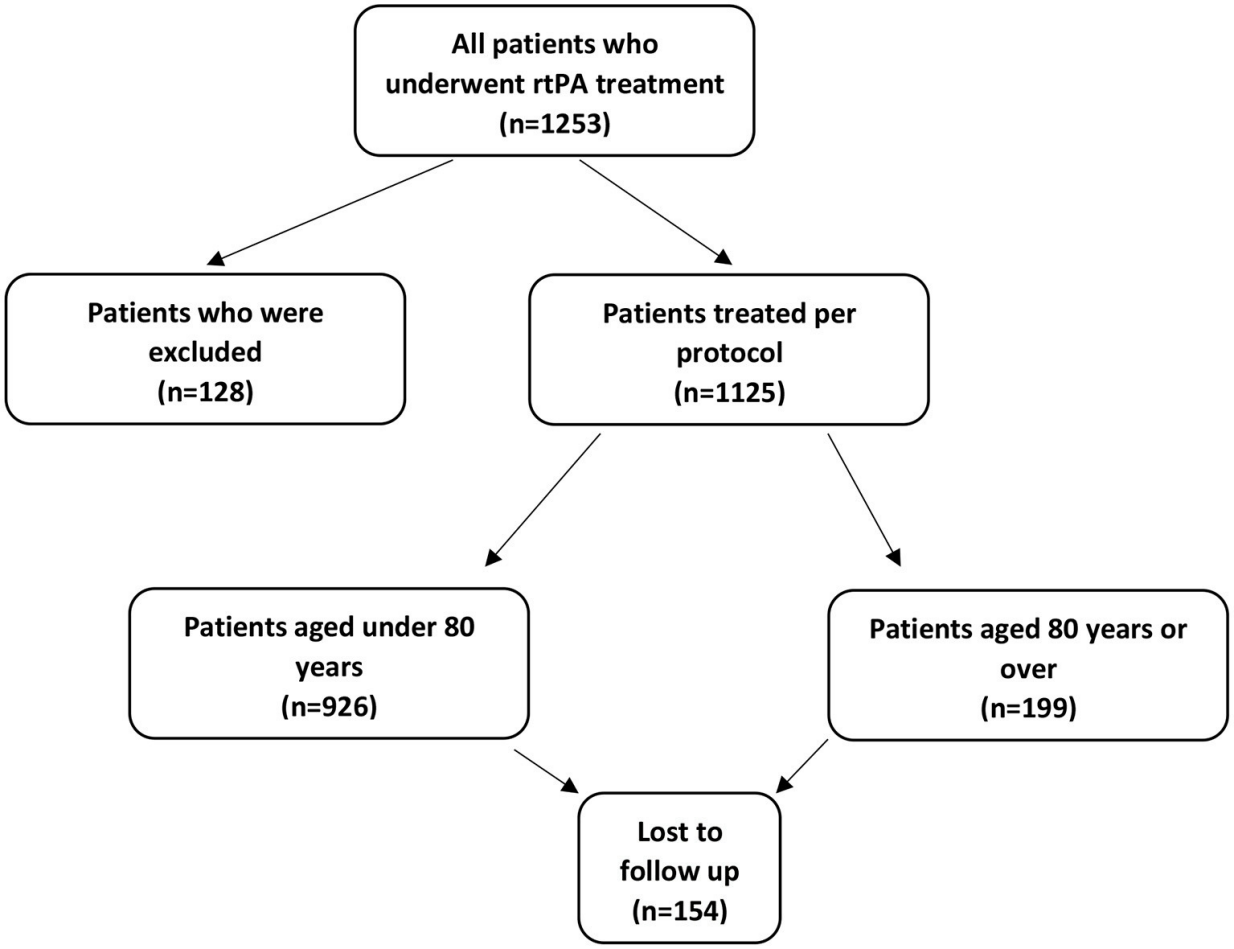
Flow chart of participants (rtPA: recombinant tissue plasminogen activator).

An analysis of 1,253 patients treated with IV-rtPA was conducted at the stroke unit of tertiary center using the thrombolysis database. The data have been collected prospectively for the period between January 1, 2004, and August 31, 2016. Patients are admitted from a 90 km radius of the center, with a catchment area of 600,000 inhabitants and 600–700 acute stroke hospitalizations per year. All the patients were treated and the parameters recommended in the ESO guideline were monitored in the Neurological Intensive Care Unit, (13–15). Some cases, where treatment indications did not follow the guidelines (13, 14), were excluded, and 1,125 patients' data were analyzed. Among them, 199 (17.6%) were aged over 80 years. From the 1,253 thrombolysed patients only 41 had mRS ≥2 before admission, six patients from ≥80 years group and 35 patients from <80 years group. These patients were excluded from further analysis. Unfortunately, 154 patients were lost during the long-term follow up (Figure 1). The patients were divided into two subgroups: patients aged under 80 years and patients aged 80 years or over. For all patients treated over 80 years, permission was obtained from the National Institute of Pharmacy and Nutrition.

### Database

The following parameters were recorded: age, gender, logistic data (stroke onset-to treatment, door-to-imaging, door-to-treatment). Risk factors for stroke, past history, hypertension, diabetes mellitus, atrial fibrillation, prestroke anticoagulation, congestive heart failure and smoking habits were also evaluated. Furthermore, all the patients were tested for blood glucose, cholesterol and triglyceride levels, as well as systolic and diastolic blood pressure on admission. Stroke severity was assessed in accordance with the National Institutes of Health Stroke Scale (NIHSS) by the neurologist currently on duty in the stroke unit on admission and 24 h later. All the patients underwent brain imaging with computed tomography on admission, at 24 h after thrombolytic therapy and in case of later neurological deteroriation. Blood pressure, laboratory parameters and onset to treatment time were expressed as mean ± standard deviation. NIHSS scores were presented as medians (1,;3. quartile).

### Imaging

Patients with acute stroke were admitted directly to the CT laboratory, where neurological examination, blood sampling and imaging were performed. Non-contrast computed tomography was performed on admission. Arterial occlusion (trunk or at least one branch of any large artery) was identified by CT-angiography. To evaluate hemorrhagic changes, CT was repeated 1 day after treatment and in case of clinical deterioration. Hemorrhagic infarction (HI) or parenchymal hematoma (PH) were defined according to the European Cooperative Acute Stroke Study (16, 17). We used three definitions for symptomatic intracerebral hemorrhage (SICH) as follows: the SITS, the ECASS and the RCT NINDS criteria (17–19). The Alberta Stroke Programme Early CT Score (ASPECTS) was determined unblinded to patient characteristics and was stratified to <7 (group I severe) and ≥7 (group II mild), within the mild group patients scoring 10 and less, were examined separately (20, 21).

### Treatment

Intravenous thrombolysis was administered according to valid guidelines (13, 14). Intravenous rtPA (0.9 mg/kg body weight, maximum 90 mg), with 10% of the dose was given as a bolus followed by a 60-min infusion. Elevated blood pressure was decreased below 185/110 mmHg according to guideline recommendations. Neurological status, side effects (allergic reactions, minor bleedings), pulse, blood pressure, temperature and oxygen saturation were monitored continuously.

### Long-Term Outcome

The modified Rankin Scale (mRS) was used to assess 3 months' outcome. The outcome was dichotomized to favorable (mRS 0-2) and unfavorable (mRS>2) points (22). At 1 year, the outcome was dichotomized to “dead” and “alive” status.

Beside these patients data, we compared the long-term outcome in patients over 80 years who underwent thrombolysis with the non-thrombolysed patients of the same age using the data from an Eastern European stroke epidemiological study, the MUD (Marosvásárhely-Ungvár-Debrecen) database (23). This database was chosen as a medical historical control. Only outcomes could be compared, since imaging workup in 1999–2000 differed from the present, and CT scans were not stored digitally.

### Statistical Analysis

Statistical analysis was carried out using the SPSS for Windows 19.0 program suite (SPSS Inc. Chicago, USA). Descriptive statistics was performed. Two-group analysis was assessed with Pearson χ^2^ test for categorical variables. For continuous variables, Mann –Whitney U test was used. The level of significance was set at *p* < 0.05. Logistic regression models were used to identify the independent predictors of 3-month disability and 1-year case fatality. The analysis was performed with the multivariate general linear model (GLM). In the models, disability at 3 months (mRS >2), and case fatality at 1 year were the dependent variables, and the factors found to be associated with outcome by univariate analyses were entered as confounding variables. The variables were excluded from the analysis one by one, and the variable with *p* > 0.05 and closest to 1.0 was removed, until all features left in the model had *p* < 0.05.

## Results

### Baseline Characteristics

The baseline characteristics of the patients are summarized in Table 1. The patients' age ranged between 17 and 99 years. The mean age of the total population was 68.2 ± 12.4 years, i.e., 64.7 ± 10.8 years and 84.3 ± 3.4 years in the younger and the older groups, respectively, (*p* < 0.001). The majority of the patients in the general population were males (56.3%). There was a significant difference in gender ratio (*p* < 0.00001): in the older group the percentage of female patients (63.3%) was higher than that in the younger group (39.5%). The risk factors for stroke differed between older and younger patients. Hypertension was the most remarkable risk factor in both groups and was more prevalent among older patients, but the difference was not statistically significant. The history of current or past smoking was the second most common risk factor (42.2%) and was significantly more likely among younger patients. Atrial fibrillation was significantly more prevalent among patients over 80 years than among younger study participants (*p* < 0.00001). Most of the younger subjects (60.9%) did not receive anticoagulation therapy before stroke, but the patients over 80 had previously been treated with oral anticoagulants much more often than in the younger ones (*p* = 0.02). Among the elderly, congestive heart failure was more common, near the significance level (*p* < 0.07) statistically. Regarding other risk factors, such as diabetes mellitus, hyperlipidemia and previous stroke, there were no significant differences between the two groups. Baseline stroke severity was significantly higher (*p* < 0.0001) among patients over 80 years than the younger ones. The median (1;3 quartile) NIHSS scores on admission being 14 (8, 18) and 10 (5, 15), respectively. Time from symptom onset to treatment did not differ significantly in the two groups.

**Table 1:** Baseline patient characteristics.

	**Age group**			
**Characteristic**	**Total**	**≥80 years**	**<80 years**	***p*-value**
	**(*n* = 1,125)**	**(*n* = 199)**	**(*n* = 926)**	
Age (years), mean ± SD	68.2 ± 12.4	84.3 ± 3.4	64.7 ± 10.8	**<0.001**
Gender, male, *n* (%)	633 (56.3)	73 (36.7)	560 (60.5)	**<0.00001**
Risk factors				
Hypertension, *n* (%)	863 (76.7)	176 (88.4)	687 (74.2)	NS
Smoking, current, *n* (%)	281 (24.9)	10 (5)	271 (29.3)	**<0.00001**
Smoking, previous, *n* (%)	138 (12.3)	18 (9)	120 (12.3)	**<0.00001**
Smoking, total, *n* (%)	420 (37.3)	28 (14)	391 (42.2)	**<0.00001**
Diabetes mellitus, *n* (%)	235 (20.9)	36 (18%)	189 (20.4)	0.062
Hyperlipidemia, *n* (%)	398 (35.3)	53 (26.6)	345 (37.3)	**<0.0001**
Atrial fibrillation, *n* (%)	198 (17.6)	69 (34.7)	129 (13.9)	**<0.0001**
Congestive heart failure, *n* (%)	153 (13.6)	35 (17.6)	118 (12.7)	0.048
Prestroke anticoagulation, *n* (%)	104 (9.2)	27 (13.6)	77 (8.4)	0.02
Vital parameters on admission				
Systolic blood pressure (mmHg), mean ± SD	156.9 ± 23	156.8 ± 25.66	157 ± 23.6	NS
Diastolic blood pressure (mmHg), mean ± SD	86.9 ± 14,4	84.3 ± 17	87.5 ± 14.2	**0.003**
Serum glucose level (mmol/l), mean ±SD	7.5 ± 2,9	7,3 ± 2,4	7.6 ± 3	**<0.0001**
Cholesterol level (mmol/l), mean ± SD	4.1 ± 2,2	3,8 ± 2,2	4.2 ± 2,2	**0.005**
Triglyceride level (mmol/l), mean ± SD	1.2 ± 1,1	1.15 ± 0,54	1.56 ± 1	**<0.00001**
NIHSS score on admission, median (1;3 quartile)	10 (6;15)	14 (8;18)	10 (5;15)	**<** **0.0001**
Onset to treatment time (min), median ± SD	158 ± 51.3	150 ± 43.2	150.5 ± 51.3	NS

### CT Characteristics

CT characteristics on admission and at 24 h compared with on admission-NIHSS, 24 h-NIHSS and 3 months-mortality in patient groups under and above 80 years are summarized in Table 2 and Table 3. Significant correlation can only be declared between ASPECT score and outcome at 24 h because of the small numbers of patients in different categories, despite the trends seen in the table. Groups were created according to ASPECT Score on admission CT scan and CT scan done at 24 h.

**Table 2:** CT characteristics of patients above and under 80 year.

	**Total**	**age ≥80 years**	**age <80 years**	**p-values**
Location of stroke				
Anterior circulation, *n* (%)	973 (86.5)	181 (91)	792 (85.5)	
Posterior circulation *n* (%)	152 (13.5)	18 (9)	134 (14.5)	
Large artery occlusion *n* (%)	619 (55)	125 (63)	494 (53.4)	0.09
ASPECT score on admission, *n* (%)				0.319
≥7		180 (99.5)	778 (98.2)	
<7		1 (0.5)	14 (1.8)	
ASPECT score at 24 h, *n* (%)				0.007
≥7		73 (40.3)	399 (50.4)	
<7		108 (59.7)	393 (49.6)	

**Table 3:** CT characteristics on admission and at 24 h compared with on admission-NIHSSS, 24 h-NIHSSS and 3 months-mortality in patient groups under and above 80 years.

	**Ratio**		**NIHSS on admission (1.;3.quartile) median**		**Ratio of mild (NIHSS 1–7) strokes on admission (pts%)**		**NIHSS at 24 h (1.;3.quartile) median**		**Ratio of mild (NIHSS 1–7) strokes at 24 h**		**3 months case fatality**	
	**≥80 years**	**<80 years**	**≥80 years**	**<80 years**	**≥80 years**	**<80 years**	**≥80 years**	**<80 years**	**≥80 years**	**<80 years**	**≥80 years**	**<80 years**
**ASPECT Score on admission**												
**10**	92.3%	90.9%	(9;18) 14	(5;14) 9	20.8%	40.8%	(4;18) 11	(3;13) 7	38.1%	53.7%	31.5%	1.2%
**9–7**	7.1%	7.3%	(10;19) 13	(7;15) 11	15.4%	28.7%	(11;20) 16	(5;16) 11	15.4%	36.7%	23%	15%
**<7**	0.5%	1.8%	(23;23) 23	(12;19) 16	0%	5.5%	(22;22) 22	(7.5;17) 12.5	0%	22.2%	100%	11.1%
**ASPECT Score at 24 hours**												
**10**	22.5%	35.3%	(6.25;14.5) 10.5	(4;10) 6	35%	65.4%	(2.75;6) 4	(1;6)3	62.5%	44.1%	10%	6%
**9–7**	18%	15.1%	(6.75;11) 18	(6;14) 9	28.1%	40.2%	(2;10.25) 13.75	(3;12)7	62.5%	56.4%	3.1%	4.7%
**<7**	59.5%	49.6%	(10.25;19) 16	(8;17) 13	10.4%	17.8%	(9;19) 15	(6;12) 17	20.7%	4.5%	46.2%	18.7%

Most strokes were located in the anterior circulation in both age groups. Probability of developing a large artery occlusion was higher among the elderly (63 vs. 53.4%), but the difference is not significant (*p* = 0.09). Most of the patients had ASPECT score ≥7 on admission in both age groups. However, median (1;3 quartile) NIHSS score on admission was higher (14 [9:18] vs. 9 [5:14]) and ratio of mild strokes (NIHSS score 1–7) was less frequent (20.8 vs. 40.8%) in the older group. At 24 h, 59.6% of patients over 80 years had ASPECT score <7 (*p* = 0.007) accompanied with higher median (1;3 quartile) NIHSS score and less ratio of milder strokes in both ASPECT group.

Nevertheless at 24 h there was an improvement, which was significant, and according to the Mann–Whitney *U* test the improvement was more pronounced in patients ≥80 than in the younger ones (*p* < 0.0001). The mean rank of on admission NIHSS Score by patients under 80 was 599.7 and at 24 h 601.5, while by patients ≥80 years on admission 755.2, at 24 h 731.75. So altogether the elderly patients had scored more on the NIHSS Scale, but the improvement was better by them.

### Outcome

**Figure 2:**
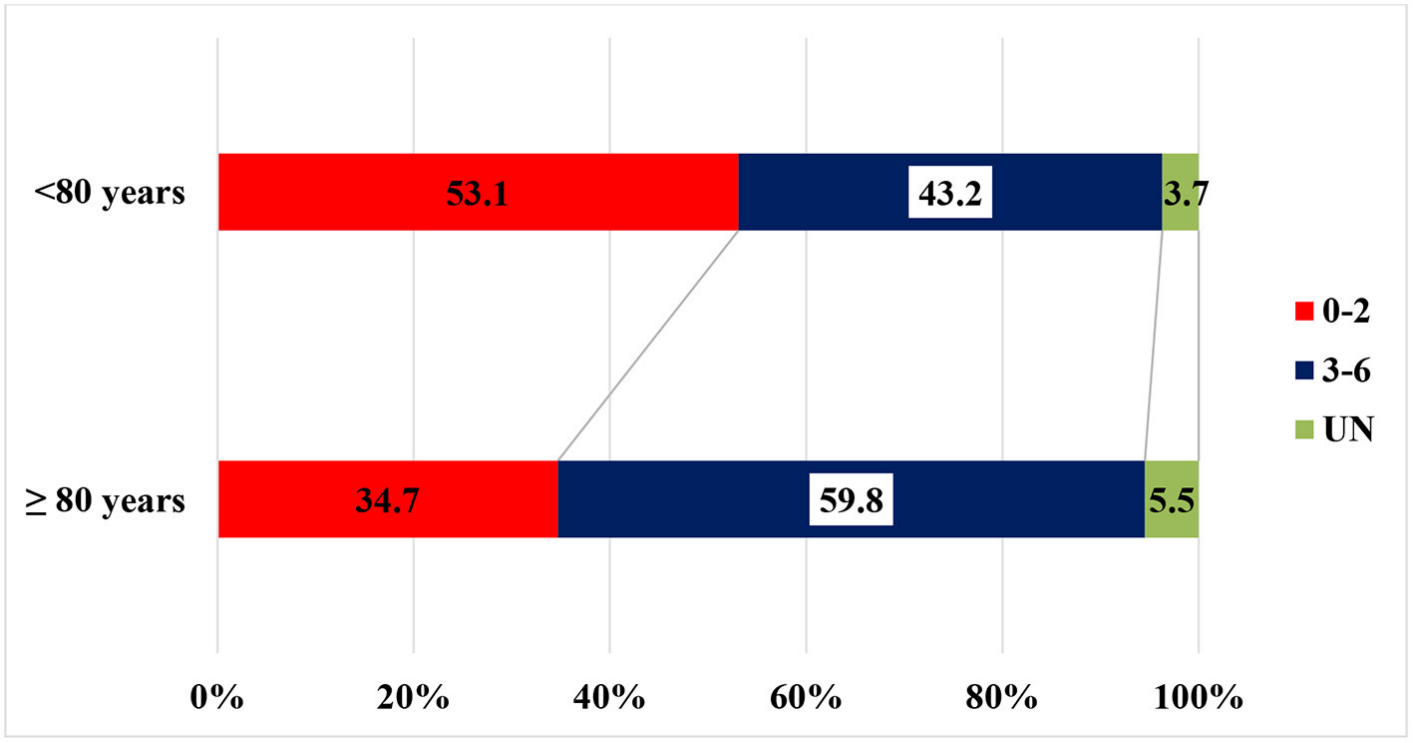
Outcome at 3 months based on mRS score.

**Figure 3:**
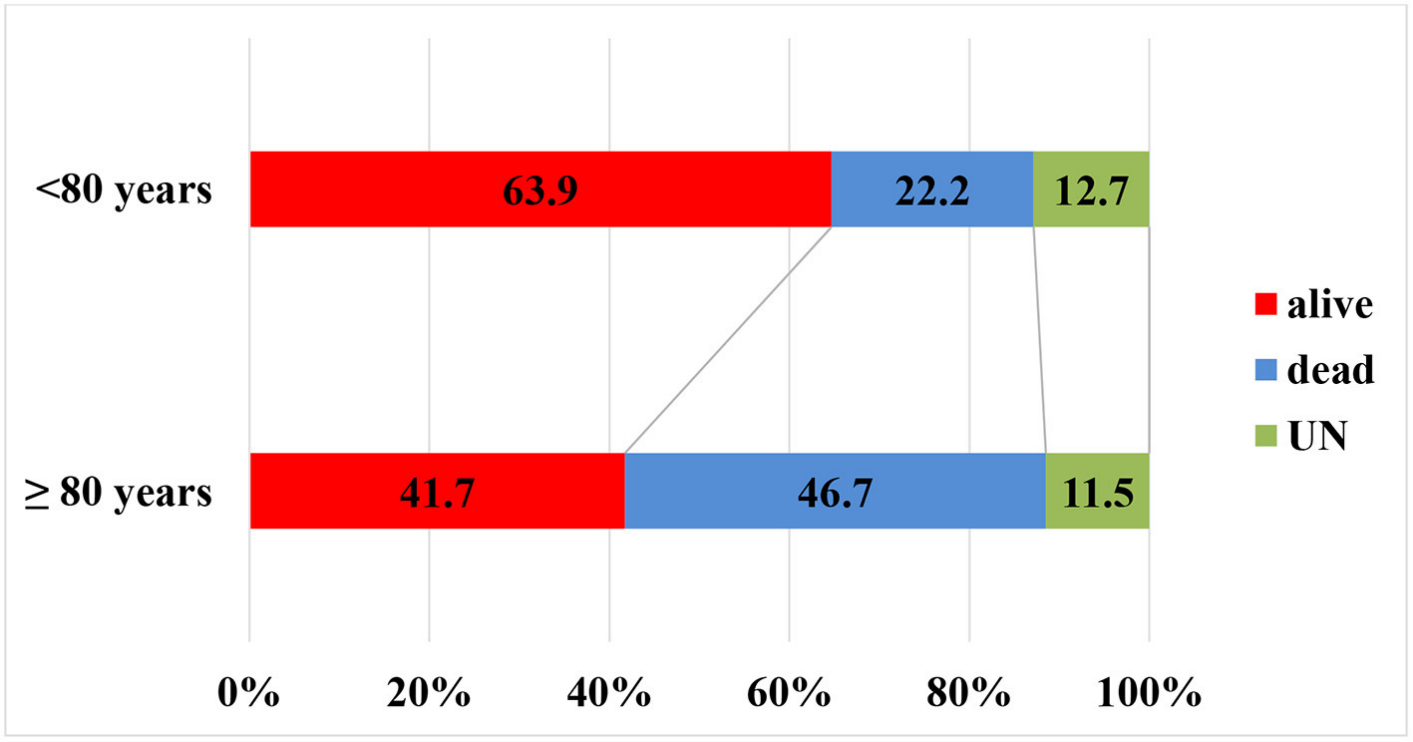
Outcome at 1 year.

Tables 4–6 and Figures 2–3 summarize the data of clinical outcomes. At 24 h, the patients over 80 had higher NIHSS scores than the ones under 80, the median (1;3 quartile) NIHSS scores seen at 11 [4.5;18] and 7 (3, 14), with the difference also being statistically significant (*p* < 0.0001). More than two points of worsening in NIHSS score were seen in 17% of patients over 80 years which is significantly worse (*p* = 0.034) deterioration compared to the younger ones (14,7%). In addition, significantly less patients (*p* = 0.00016) achieved at least two points improvement in NIHSS score over the age of 80 compared to the younger group (39.7 and 47.4%, respectively) (Table 4) At 3 months, 59.8% of the patients in the older group had unfavorable outcomes (mRS: 3-6), which was significantly worse (*p* < 0.0001) compared to the younger age group (43.2%). However, 34.7% of the patients over 80 had independent outcomes (mRS: 0-2), and more than two thirds of them were able to continue their pre-stroke activities (mRS: 0-1). There was an unfavorable trend regarding the 90-day outcomes for the over-80 patients treated for atrial fibrillation compared to patients of the same age group without atrial fibrillation. While in the former group 27.7% of patients had favorable outcomes, the particular proportion was 41.5%, among the patients without atrial fibrillation. The difference was not significant. The one-year survival in the older group was 41.7%, while the proportion in the younger group was 63.9%, the difference being statistically significant.

**Table 4:** Median NIHSS scores and changes in NIHSS at 24 h in patient groups above and under 80 years.

	**age ≥80 years**	**age <80 years**	***p*-values**
NIHSS score at 24 h, median (1;3 quartile)	11 (4.5;18)	7 (3;14)	***<0.0001***
Changes in NIHSS score at 24 h			
>2 points worsening, *n* (%)	34 (17)	136 (14.7)	***0.034***
≤2 points worsening, *n* (%)	25 (12.6)	99 (10.7)	0.27
unchanged, *n* (%)	40 (20.1)	160 (17.3)	0.19
≥2 points improvement, *n* (%)	79 (39.7)	439 (47.4)	***0.00016***
<2 points improvement, *n* (%)	21 (10.6)	92 (9.9)	0.38

**Table 5:** mRS score at 3 months and 1-year mortality in patient groups above and under 80 years.

		**Age group**			
	**Total**	**≥80 years**	**<80 years**	**Missing data, *n* (%)**	***p*-value**
mRS score at 3 months					**<0.0001**
Favorable outcome (mRS:0–2), *n* (%)	561 (49.9)	69 (34.7)	492 (53.1)	48 (4.2)	
Moderate disability (mRS:3–4), *n* (%)	222 (19.7)	31 (15.6)	191 (20.6)	48 (4.2)	
Severe disability/death (mRS:5–6), *n* (%)	297 (26.4)	88 (44.2)	209 (29.6)	48 (4.2)	
Mortality at 1 year	299 (26.6)	93 (199)	206 (22.2)	154 (13.7)	**<0.0001**

**Table 6:** Predictor of outcome with logistic regression mode.

**Predictor of outcome**			**Predictor of outcome**		
**<80 years**			**over 80 years**		
**Disability at 3 months**					
	**Exp(B) (95% CI)**	**p**		**Exp(B) (95% CI)**	***p***
Atrial fibrillation	0.524	**0.001**	**atrial fibrillation**	0.550	**<0.001**
	(0.363-0.756)			(0.396-0.763)	
Heart failure	0.547	**0.004**	**heart failure**	0.033	**0.014**
	(0.362-0.826)			(0.44-0.911)	
			**diabetes mellitus**	0.725	**0.037**
				(0.536; 0.981)	
**Survival at 1 year**					
Diabetes mellitus	0.705	**0.042**	**None**		
	(0.504;0.988)				
Actual smoking	0.585	**<0.001**	**None**		
	(0.432;0.791)				

In a logistic regression model of the patients under 80 years, atrial fibrillation and heart failure were significant independent risk factors for worse outcomes at 3 months, whereas among the elderly subjects diabetes mellitus was also a risk factor for a worse outcome (Table 5). At 1 year, smoking and diabetes mellitus were significant risk factors in the younger group, while no independent risk factor was found among the elderly at 1 year follow-up (Table 6).

We compared the mortality rates for the patients over 80 years of age having undergone thrombolysis, to the MUD (Marosvásárhely-Ungvár-Debrecen / Târgu Mureş - Uzhhorod - Debrecen) database's non-thrombolyzed patients of the same age at 3 months and 1 year. The results are as follows: at 3 months, in the over-80-group, the mortality rate was 30.1%, and in the under-80 it was 33.3% while, at 1 year, the relevant figures were 46.7 and 56.8%, respectively), but the difference was not significant.

### Safety

Regarding the hemorrhagic complications of thrombolytic therapy, intracranial hemorrhage occurred in 125 patients (9%), while SICH was detected in 36 patients (3.2%). Intracranial hemorrhage and SICH did not differ significantly between the elder and younger patients (Table 7).

**Table 7:** Occurrence of intracranial hemorrhage and symptomatic intracerebral hemorrhage in patient groups under and above 80 years.

	**Total**	**Age ≥80 years**	**Age <80 years**	***p*-values**
ICH, *n* (%)	125 (9%)	19 (9.4%)	106 (11.5%)	0.28
SICH, *n* (%)	36 (3.2%)	4 (2.1%)	32 (3.5%)	0.25

## Discussion

As life expectancy increases, the proportion of the elderly population is constantly growing. In absolute terms, the number of older persons has doubled over the last 20 years and will more than triple again over the next 30 years (24). The aging society puts a heavy burden on healthcare, not sparing the stroke care either, especially in very old age. Though, more and more studies come to light on the safety of IV-rtPA treatment in acute ischemic stroke among patients over 80 years, the uncertainty still exists in clinical practice. In this single center study, we analyzed the data of 1,125 patients who underwent intravenous thrombolysis, comparing the baseline characteristics and clinical outcomes between patients over and under 80 years.

Consistent with previous studies (25–28), our findings showed that hypertension was the most important risk factor in both subgroups, and it was more prevalent in older patients. Therefore our study points out that optimizing antihypertensive treatment and maintaining blood pressure below the target level may lower the risk for stroke. As described in previous studies (29, 30), older patients were significantly more likely to develop atrial fibrillation than the younger ones. Atrial fibrillation is associated with a five-fold increase in the risk for ischemic stroke, but anticoagulant therapy may reduce the risk of recurrent stroke by ~by 60% (31, 32). Despite the finding that more than one-third of the patients over 80 years had atrial fibrillation, only 13.6% were previously medicated with oral anticoagulants. Atrial fibrillation was a significant prognostic factor for more severe functional status in univariate model. More detailed in a logistic regression model, there was a difference between the risk factors among patients under and over 80 years. Atrial fibrillation and heart failure were significant independent risk factors for worse outcomes at 3 months among the younger subjects, whereas diabetes mellitus was also a risk for worse outcome among the elderly. At 1 year, smoking and diabetes mellitus were significant risk factors in the younger group, while no independent risk factor was found among the elderly at 1 year follow-up. These findings suggest that by proactively searching for atrial fibrillation and providing effective anticoagulant therapy, the risk of stroke can be reduced and better functional outcome can be achieved. Regarding other vascular risk factors, the prevalence of diabetes and hyperlipidemia did not show significant differences between the two groups, but the history of current or past smoking was more common in younger age. These findings suggest that changing unhealthy lifestyles is of great importance in the prevention of ischemic stroke in both age groups.

Multiple studies have proven that both admission and 24-h stroke severity are poor prognostic factors for long-term outcome (7, 33). An important result of our study shows that older patients tend to experience stroke of higher severity than younger patients previously reported (median NIHSS score of 14 vs. 10), (33, 34). Furthermore, patients over 80 years of age also had significantly higher NIHSS scores at 24 h than younger patients did. Nevertheless according to the Mann–Whitney *U* test, by the elderly patients the improvement was better. These results suggest that thrombolytic therapy has a positive effect in patients above 80 years.

Regarding the correlation of CT parameters and outcome, we found that on admission ASPECT score was similar in the groups of patients above and under 80 years (*p* = 0.319), but despite this the on admission NIHSS score was higher in the elderly and less patients had milder strokes on admission. Large artery occlusion was more frequent in the elderly (*p* = 0.09), and although there was no sign of hyperacute ischemia, it had an effect on ASPECT at 24 h. ASPECT score at 24 h was less favorable in elderly patients (*p* = 0.007). Not surprisingly with the help of 24 h- ASPECT Score prognosis could be estimated closer than with the on-admission ASPECT Score. Interestingly the ratio of mild strokes at 24 h is less in ASPECT Score <7 by younger patients. This might emphasize the importance of functional collaterals in older ones. Analyzing the 3 months mortality, a higher rate can be detected among elderly patients especially, if the ASPECT Score is <7 points, younger patients have a better chance to survive at 3 months even with more severe CT abnormalities.

As for the long-term outcome, the functional status at 3 months turned out to be significantly worse in the older age group. This result is consistent with most of the recent studies which have shown lower rates of favorable and independent outcomes at day 90 among patients over 80 years of age (35–37). Despite the above, older patients still benefited from IV-rtPA because more than one-third of them were able to live independently. It should also be noted that the modified Rankin Scale not only estimates function loss due to stroke, but it the prevalence of disability increases with age, regardless of stroke (38). Although in our study prestroke disability did not differ in different age groups, but if the on admission-ASPECT Score and/or 24-h ASPECT Score was <7, the case fatality was extremely worse in the elderly patients. Similarly, the one-year mortality rate does not only reflect deaths due to stroke, as its prevalence increases with age regardless of stroke (39). The comparison of our results with the MUD database also supports the safety of thrombolysis because the mortality rates do not differ among over 80-year-old patients after thrombolysis compared with the non-thrombolyzed patients of the same age.

The occurrence of the ICH, SICH was similar in patients below and above 80 years. This is especially important, if we consider that the ratio of the large artery occlusion was more frequent in patients above 80 years. This confirms the safety of thrombolysis in elderly patients. Concerning the risk of therapy, in our study SICH occurred in 3.2% which is somewhat less than reported in major rtPA trials (NINDS 6.4%, ECASS II 8.9%) (17, 18). A hypothesized higher risk for intracerebral hemorrhage is often cited as the reason for excluding very old patients from thrombolytic treatment. Amyloid angiopathy, decreased renal rtPA clearance, and frail vasculature in the elderly are asserted as explanations for a possibly increased risk of suffering an intracerebral hemorrhage (40). However, many other studies had previously reported that the occurrence of SICH did not differ significantly between younger and older groups of patients (7, 41–43). Our results are in line with these studies as we have found that the prevalence of SICH tended to be lower in older than younger patients (2.1 and 3.5% respectively, not significant). Further investigation is needed to determine the underlying cause.

Of course, we are aware of the limitations of our study. Although the number of study participants is small, the most important and relevant risk factors have been identified. Nevertheless, the advantage is the real-world scenario.

In conclusion, according to our results, patients with acute ischemic stroke and over 80 years seem to have an increased risk for unfavorable outcome and a higher mortality rate compared to their younger counterparts. However, intravenous thrombolysis is an effective and safe treatment in this age group, as more than one-third of the patients were capable of living independently and the rate of SICH was lower compared to younger patients. Although, the outcomes were less favorable in patients over 80 years of age, our results support the feasibility of using intravenous thrombolysis among patients over 80 years of age. Consequently, these data support that age by itself should not be a reason to exclude patients over 80 years old from IV-rtPA treatment.

## Data Availability Statement

The raw data supporting the conclusions of this article will be made available by the authors, without undue reservation.

## Ethics Statement

The studies involving human participants were reviewed and approved by the Regional and Institutional Ethics Committee of University of Debrecen Clinical Center (protocol number: 5473-2020). Written informed consent for participation was not required for this study in accordance with the national legislation and the institutional requirements.

## Author Contributions

KF, IF, and LH led the initiative and revised the drafted document. KF, LH, and MH selected abstract, extracted data, and drafted the manuscript. SM, LH, KF, and MH is involved in investigation, data curation, data analysis, and writing the original draft. IF and KF were involved in supervision. All authors are involved in the conceptualization, methodology, review and editing, and approved the final version.

## Conflict of Interest

The authors declare that the research was conducted in the absence of any commercial or financial relationships that could be construed as a potential conflict of interest.

## Publisher's Note

All claims expressed in this article are solely those of the authors and do not necessarily represent those of their affiliated organizations, or those of the publisher, the editors and the reviewers. Any product that may be evaluated in this article, or claim that may be made by its manufacturer, is not guaranteed or endorsed by the publisher.
